# Involving End Users in Co-Designing Mobile Health Interventions for Hypertension Self-Management: Formative Study

**DOI:** 10.2196/77631

**Published:** 2026-01-21

**Authors:** Amber Johnson, Priya Nair, Deekshita Behara, Alexis Aranda-Hernadez, Jamil Bey, Christina N Harrington, Elizabeth Miller, Monica E Peek, Spyros Kitsiou, Jared W Magnani

**Affiliations:** 1 School of Medicine, University of Chicago, 5841 South Maryland Avenue, Chicago, IL, 60637, United States, 1 773-702-7769, 1 773-702-8875; 2 Biological Sciences Division, Section of Cardiology, School of Medicine, University of Chicago, Chicago, IL, United States; 3 School of Medicine, University of Pittsburgh, Pittsburgh, PA, United States; 4 Illinois Wesleyan University, Chicago, IL, United States; 5 UrbanKind Institute, Pittsburgh, PA, United States; 6 Human-Computer Interaction Institute, School of Computer Science, Carnegie Mellon University, Pittsburgh, PA, United States; 7 Adolescent and Young Adult Medicine, Department of Pediatrics, University of Pittsburgh, Pittsburgh, PA, United States; 8 College of Applied Health Sciences, Department of Biomedical and Health Information Sciences, University of Illinois Chicago, Chicago, IL, United States

**Keywords:** community-engaged research, hypertension, intervention design, human-centered design, mobile health, mHealth

## Abstract

**Background:**

Mobile health (mHealth) interventions are prevalent, yet people from marginalized communities are less likely to use digital health technologies to support self-management behaviors. Community engagement can inform health care design to enhance a hypertension self-management mHealth intervention.

**Objective:**

We applied human-centered design (HCD) to determine appropriate iterations of an existing hypertension intervention.

**Methods:**

Through an equity-focused, community-centered approach, we strove to optimize an mHealth app. We used validated theories and frameworks as well as an HCD methodology organized into three fundamental design skills: (1) methods to directly observe user experiences, (2) methods to analyze barriers to ideal intervention use, and (3) methods to design future iterations.

**Results:**

In October 2023, we conducted a series of HCD activities with a community advisory board (n=8) to refine an mHealth intervention for hypertension. Participants tested app prototypes with blood pressure monitors and suggested content modifications to enhance intervention fidelity. Among 6 participants, usability testing scored 67.5 (benchmark 68, “above average”), with all users finding the tool easy to use. Feedback identified critical needs, barriers, and work-arounds for future mHealth iterations.

**Conclusions:**

This study was a novel use case example of HCD as a patient-centered methodology to improve a hypertension management tool.

## Introduction

Hypertension is a public health crisis and is a leading modifiable cause of cardiovascular disease (CVD) worldwide [1,2]. Control of hypertension in the United States plateaued over a decade ago, and current recommendations of blood pressure (BP) of less than 130/80 mm Hg are achieved by only 19% of patients [3-5]. Furthermore, populations experiencing social and structural inequities (eg, due to race) face significant disparities in clinical outcomes [6]. Race-based differences in hypertension self-management may exacerbate preventable health disparities in stroke, kidney disease, atherosclerosis, and mortality [7]. Professional society guidelines prioritize nonpharmacological and behavioral interventions for adults with elevated BP and hypertension and advocate for implementation strategies to overcome race-based disparities [8,9]. Behavioral strategies for monitoring and managing BP can moderate the progression of hypertension and potentially address disparities.

Newer research has shown that mobile health (mHealth) technologies can yield meaningful health behavior change. Technology-based behavior change interventions can demonstrate measurable and clinically meaningful effects. When used for chronic disease management practices, they can be engaging and easily adopted in some patient populations [10]. Successful self-management technologies can include individualized, patient-facing content [11,12], such as mHealth virtual coaches that provide lifelike, digital education, monitoring, and problem-solving [13-17]. mHealth has emerged as a scalable technological advance to support behavior change for hypertension self-management [18,19]. The popularity of mHealth for hypertension and CVD management is rising in part because smartphone use has become nearly ubiquitous [20]. However, a divide in health care and technology access across race, ethnicity, economic status, geography, and education levels remains [21], potentially perpetuating health care inequity [22,23]. Despite the prevalence of mHealth interventions, people from marginalized communities, including Black people, are less likely to use digital health technology to support self-management behaviors [24,25]. Community engagement [26] and community-based approaches can be important when tailoring technological interventions to meet the needs of unique patient populations [27,28].

Self-management behaviors for hypertension are central to the prevention of comorbid disease [29]. The targeted behaviors should include home BP monitoring, medication adherence, and lifestyle modifications such as healthy nutrition and physical activity [30]. Our group’s preliminary mHealth work demonstrated the usefulness of a bespoke virtual mHealth coach among participants with CVD. In a pilot study (n=120), our group showed that our mHealth tool was feasible, acceptable, and engaging for participants, the majority of whom were White with moderate incomes [19]. Although this intervention was designed to be patient centered, preliminary testing had not been conducted among a diverse patient population. What is unknown is whether and how such tools can be useful and optimized in sociodemographically diverse populations [10,31]. Optimizing our existing mHealth tool for hypertension will allow further development using validated, patient-centered, theory-informed behavioral methodology among racially minoritized populations, including Black patients. This study was part of a larger research project designed to inform hypertension management for an underserved patient population. The overall research goal was to empower individuals from marginalized backgrounds to develop an acceptable technological tool for hypertension self-management. The project was informed by a community advisory board (CAB) as a validated approach to intervention design. We used human-centered design (HCD) to engage the CAB in testing the existing mHealth tool and guide iterative changes [32]. To supplement our HCD activities, we used the validated Framework for Reporting Adaptations and Modification to Evidence-Based Interventions (FRAME) model to document details of proposed modifications to track when, how, and why modifications were being suggested [33]. Through our community-informed HCD approach, we sought to ensure that our intervention captured the needs, preferences, and capabilities of our population of focus. Thus, in this study, we hypothesized that through an iterative, community-informed approach, we could enhance a hypertension mHealth self-management intervention to facilitate use among racially minoritized patients at risk for hypertension disparities.

## Methods

### Group Members

Community partners and people living with hypertension were integral to guiding this user-centered process. Community-based methods have been described by others as critical to designing interventions that are informed by feedback from community partners, including people with the condition of interest, advocates, and community leaders [34]. AJ, EM, and JB had previously worked together to establish a CAB that would guide research goals, including the HCD activities, dissemination of research findings, and participation in community events. The CAB included racially diverse individuals from Black, White, and Asian backgrounds. Several CAB members had self-described hypertension or CVD. They resided in urban communities with high economic adversity and included community leaders involved in neighborhood-level transformation. Eight CAB members participated in the HCD activities described herein. The participants were included through convenience sampling based on their availability to attend the HCD session. Table 1 provides details about the HCD session participants.

**Table 1.** Session attendees.

Participant	Expertise
21-y-old Black man	Community activist and has participated in HCD^a^ previously
21-y-old Black woman	Master’s student in public health
60-y-old Black man	Living with chronic illness and has hypertension
38-y-old Black woman	Works in health professions and has parents with hypertension
57-y-old White man	Retired primary school teacher in disenfranchised school system and has hypertension
35-y-old Black man	Living in a racially segregated neighborhood and has hypertension
19-y-old Asian woman	Health professions student and has parents with chronic illness
52-y-old Black man	Community activist and has hypertension

a HCD: human-centered design.

### The Existing mHealth Tool

**Figure 1.**
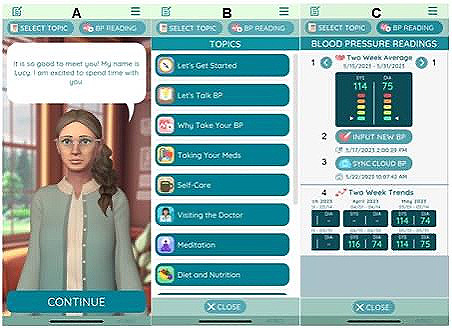
Example screenshots of the intervention*.* The app is a virtual coaching platform that allows users to perform blood pressure measurements via Bluetooth. Users can view readings and historical data. Educational modules feature several topics relevant to hypertension management and lifestyle modification. (A) “Conversation” with the coach in which she introduces herself to the patient. (B) Menu of educational topics. (C) Example patient data with 2-wk trends of recent blood pressure readings.

Our mHealth virtual tool facilitates human-device interaction and is delivered via a smartphone app. The program incorporates one-on-one coaching and education modules to support behavior change, self-management, medication adherence, and improved quality of life. The patient engages with the smartphone app by selecting options on the touch screen as shown in Figure 1. By choosing responses, patients “converse” with a virtual health coach and develop a therapeutic alliance. The program can record responses to simulate a 2-way conversation. As patient self-monitoring works best when paired with counseling and education [35], our mHealth tool was created to integrate home BP monitor use with educational components to teach patients how to track their BP data. The intervention tested in the present study included (1) patient-centered strategies to address barriers to treatment adherence, (2) disease-specific education, and (3) self-management strategies, including guided physical exercise, heart-healthy diet, and stress mitigation.

### HCD Activities

We applied HCD to determine appropriate iterations of the existing intervention that would encourage lifestyle modifications and self-management behaviors among Black patients with hypertension. Through an equity-focused, community-centered approach, we strove to optimize an existing intervention to achieve participant consensus [36]. Table 2 includes the HCD elements incorporated into our theory-driven approach. The LUMA Institute is a design firm specializing in a “practical, flexible and versatile approach” to HCD methodology [37]. Their HCD process includes a proprietary set of 36 methods organized into three fundamental design skills: (1) looking methods to directly observe user experiences in real time; (2) understanding methods to analyze barriers to ideal intervention use; and (3) making methods to design future iterations [37]. We sequentially used activities from each of the 3 groups of fundamental skills to encourage a thorough, multifaceted discussion. The sessions were moderated by AJ, a LUMA-certified HCD practitioner.

Our goal was to take the existing mHealth tool and progressively apply HCD methods, beginning with looking methods. The “that’s on your radar” activity encouraged session participants to categorize and rank the essential requirements for hypertension management. Next, “think-aloud testing” allowed participants to envision ways BP monitoring can be improved while audibly describing the process of using the intervention. This step was an opportunity to reveal flaws or inefficiencies in the mHealth tool design. The third looking method, “critiquing the system,” was built upon think-aloud testing by providing a forum for constructive feedback. Participants critically appraised the current ways in which hypertension is managed with our existing tool, with particular attention to barriers to and facilitators of self-management. We followed the critiquing step with the validated System Usability Scale [38] to score key aspects, including ease of use, technical difficulty, and one’s confidence in using the tool. The System Usability Scale includes 10 questions answered on a Likert scale, ranging from 1 (strongly disagree) to 5 (strongly agree) [38]. Survey responses were deidentified. Our next activity was an understanding method to frame participants’ experiences with hypertension management, called the “importance and difficulty matrix.” Another understanding method, “rose, thorn, bud,” encouraged participants to describe aspects as positive, negative, or having potential (a rose, thorn, or bud, respectively). Finally, we used the “cover story” activity as our making method for envisioning future possibilities of hypertension management.

**Figure 2.**
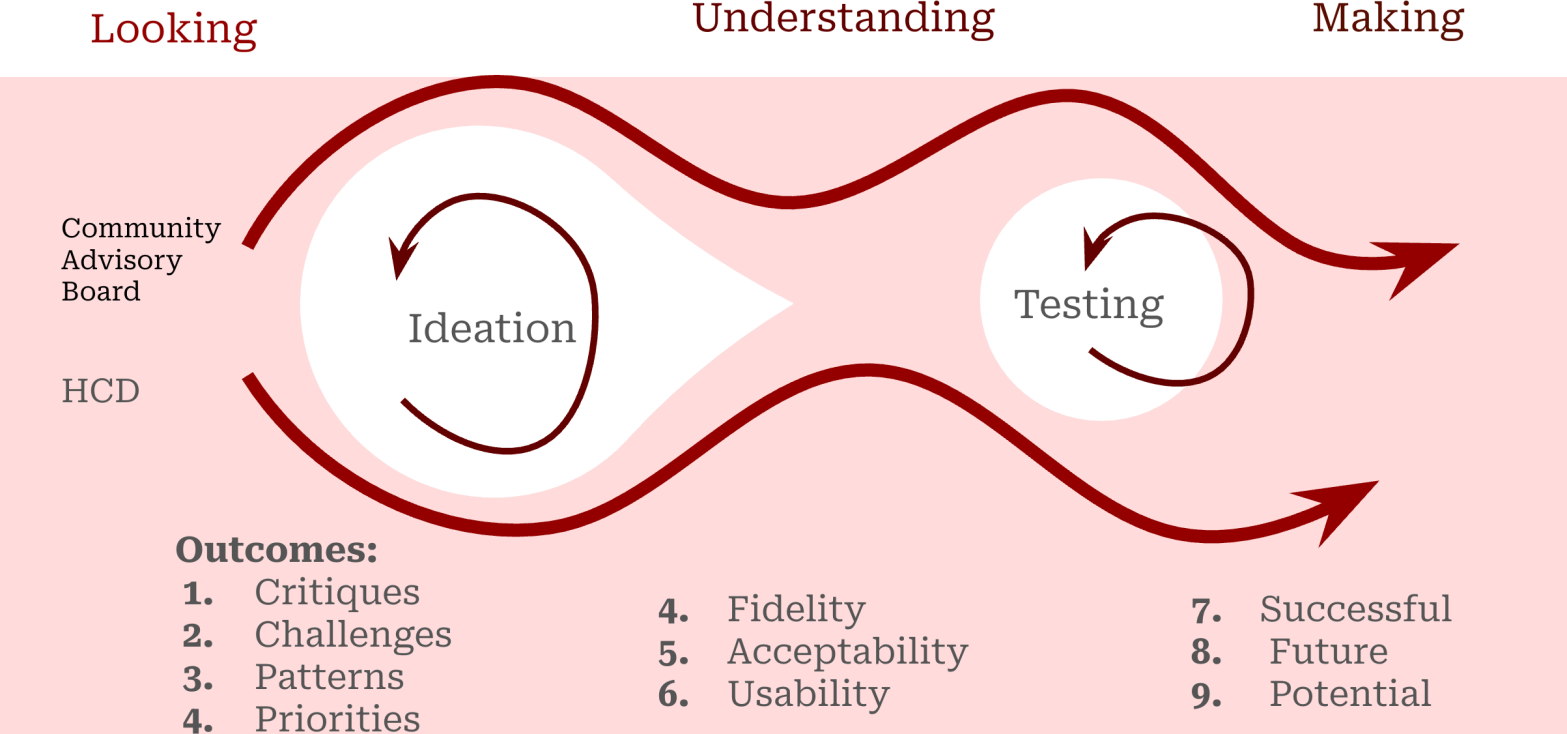
Human-centered design (HCD) process. This figure depicts the human design workflow using LUMA’s looking, understanding, and making methods in which the intervention underwent critiques from the community advisory board members. Outcomes of the ideation and testing process included narrative descriptions of patterns, qualitative assessment of priorities, and appreciation of intervention usability. The end result was a description of a potential future successful hypertension intervention for this patient population.

Attendees had access to working BP monitors and a beta version of the intervention app on study smartphones. Session activities are described in Table 2, in which relevant FRAME process elements are also explained. The HCD process allowed the group to progressively work through each specially designed activity with naturally occurring moments of ideation, wherein thoughts were discussed and elaborated upon. Additionally, the process allowed the group to concentrate on concepts that required deeper exploration or more intense focus while testing out the tool in real time. Figure 2 visually represents the HCD process for ideation, testing, and optimizing an effective hypertension management tool.

**Table 2.** Session activities.

Activity	HCD^a^ method	Possible adaptations and modifications	Emergent themes relating to self-care
What’s on your radar	Looking method Encourages participants to creatively describe the scope of a situation (in this case, BP^b^ management)	Facilitated discussions of the contexts in which one manages BP Aspects of BP management most related to fidelity were plotted in the center of the diagram Proposed modifications to the intervention included categorization into 1 of 5 topics: patient-related factors, clinician-related factors, access to health care or mHealth^c^, behavior, and technology use Significant components of BP management included self-care, education, and behavior change	Positive and negative attitudes about physical activity Negative attitudes about the health care industry and patient-physician relationships Beliefs about one’s ability to control health behaviors including sleep, diet, physical activity, and mental health
Think-aloud testing	Looking method Encourages participants to evaluate a process and provide constructive feedback Session was audio recorded	Use of intervention prototype facilitated discussions of possible content modifications Participants overall found the process of BP monitoring satisfactory Proposed modifications included cultural adaptations (what the virtual coach looks like) Suggested modifications regarding cost and access (making the tool affordable and accessible) were discussed	Positive attitudes about self-care Norms about self-care Beliefs and perceived power Related being able to control one’s BP Perceived power about education through the mHealth tool versus other modalities
Critiquing the system	Looking method Encourages participants to critique the system or process Participants completed a survey to assess usability	Survey results revealed that improvements could be made to module integration All but 1 participant found the intervention cumbersome to use	Participants were asked if other people would be able to learn to use this system very quickly: responses were neutral Thinking of others who would use this mHealth tool could normalize BP self-care behaviors
Importance and difficulty matrix	Understanding method Allows for thoughtful framing and understanding of patterns and priorities about BP management	Facilitated discussion of how important or difficult modifications to the mHealth intervention might be. Modifications that were both important and difficult were considered strategic priorities for future mHealth interventions: Building trust in technology, actualizing what one’s BP numbers mean, and understanding how behaviors affect BP Accessing the mHealth tool and improving knowledge were considered high-value modifications	Subjective norms were emphasized, including the need for behavior change and stress mitigation Perceived power was reflected in the participants’ view that BP management should become habitual
Rose, thorn, bud	Understanding method Allows participants to understand and characterize BP management Technique identified factors of the intervention as positive, negative, or having potential	Process of BP monitoring could be modified so that self-management is less of a hassle The intervention could be modified to provide direct feedback on whether the BP readings are normal and how to respond to abnormal results Potential modifications (eg, increased educational modules) to mHealth would help overcome health disparities	Positive attitudes included improving BP for a healthier, longer life Having hypertension is common and could be addressed at the community level Control beliefs regarding abnormal BP readings often lead to uncertainty or fear Perceived power was described regarding being able to take action on one’s hypertension
Cover story	Making Method Encourages the design of a future tool with discussion of the required actions for the intervention to thrive	Modifications (eg, facilitation of blood pressure management with clinicians) for successful future of BP management could lead to thriving communities and health equity	Leading heart-healthy lifestyles has implications for neighborhoods as well as individuals mHealth tools could facilitate health equity, which would in turn lead to longer, happier lives Positive findings would benefit society

a HCD: human-centered design.

b BP: blood pressure.

c mHealth: mobile health.

### Theory-Informed Intervention Assessment

In addition to the HCD activities, we used validated frameworks to complement the assessment of the end user experience. The FRAME method includes documenting details of proposed modifications to track when, how, and why modifications are being suggested [33]. We were particularly interested in modifications relevant to digital equity in mHealth interventions, including increased accessibility and usability [25]. Additionally, we used the Theory of Planned Behavior to conceptualize evidence-based, clinically meaningful behavior change techniques to enhance the intervention. Compared to other theories used to guide mHealth interventions for chronic disease self-management, the Theory of Planned Behavior features validated constructs for designing, implementing, and evaluating interventions and has been shown to be effective in hypertension management [39,40]. Our work was also informed by the recommended health behaviors identified in the American Heart Association’s Life’s Essential Eight schema, including physical activity and a heart-healthy diet [41]. These frameworks facilitated our understanding of possible ways to optimize our intervention.

### Anticipated Observations

As this was a formative process, we hoped to gain important insight for future iterations of the mHealth intervention. Direct observation allowed the session leaders to document the interplay of people, objects, and tools while completing the session activities. Therefore, target outcomes included observations of processes and themes that would be used to enhance future design efforts. Parts of the sessions were photographed or audio recorded and transcribed. The goals of our observations were to understand needs, barriers, and work-arounds to be optimized in future intervention design.

### Data Analysis

We used qualitative methodology to interpret the study observations. Audio data were recorded using a digital recording device, transcribed verbatim, and coded by AJ. Confidentiality, transparency, and verbal consent to record were established before activities. Deductive coding summarized qualitative themes based on the overarching research goals as reflected in the HCD methodology. During coding, unique observations were added to the predefined categories, such that the analysis also allowed for an inductive analytical process where appropriate. Summarized data across all activities were entered into a single matrix organized by possible future intervention iterations and self-care goals. Co-authors met to review the matrix, identify themes and unique insights across activities, and contextualize themes for clinical relevance and design implications. Disagreements were adjudicated until consensus was reached. Additionally, co-authors reviewed figures to ensure transparency and accuracy based on the HCD activities that occurred. Descriptive data are listed, and we provide the calculated usability survey results, including mean scores, SDs, and total scores for each respondent.

### Ethical Considerations

This study underwent ethical review by the University of Pittsburgh Institutional Review Board and was determined not to be human subjects research, and therefore, informed consent was not required. CAB members were compensated US $500 after their 12-month involvement with the advisory board’s overarching research program. For CAB member privacy and confidentiality, data were deidentified prior to analysis as a protective measure to safeguard personal information.

## Results

### Group Members

In October 2023, we met to conduct HCD activities for optimization of the hypertension management mHealth intervention. The meeting’s 8 attendees were CAB members aged 19 to 60 years and included 3 (38%) women and 5 (63%) men. Six (75%) of the attendees self-identified racially as Black, and all self-identified as advocates for health concerns related to the Black community (Table 1).

### HCD Activities

#### Looking: Methods to Observe User Experiences

**Figure 3.**
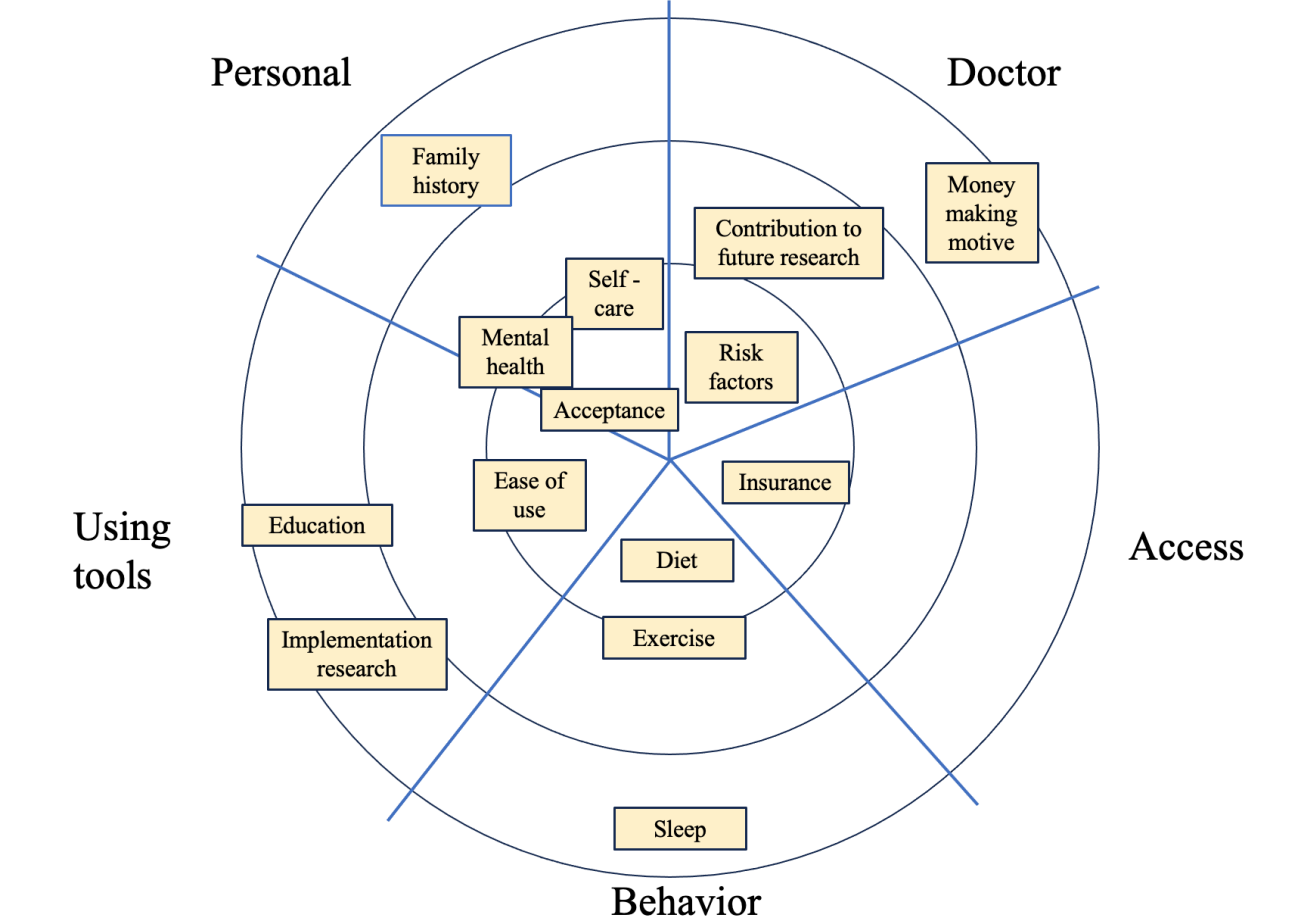
Looking activity—What’s on your radar? Participants categorized components of hypertension management and prioritized them as being of high, medium, or low importance.

The first activity, “what’s on your radar?” encouraged participants to contextualize important aspects of BP monitoring and hypertension self-management as shown in Figure 3. Participants categorized components of hypertension management according to their relevant contexts, such as patient-related behavioral aspects, including eating a healthy diet. Components of hypertension management were also prioritized as being of high, medium, or low importance.

In the next activity, “think-aloud testing,” participants used prototypes of the intervention and were able to narrate potential modifications to the intervention’s content, interface, and operation. This session was audio recorded and transcribed to assess themes of observed user experiences. All proposed modifications were proactive, meaning that they would be optimizations to the existing mHealth tool, thereby encouraging intervention fidelity [33]. Using the Theory of Planned Behavior, participants’ attitudes, subjective norms, control beliefs, and perceived power were elicited through guided questions from the session moderator (AJ) [39]. Table 3 includes quotes from the recorded session with connections to possible mHealth design elements.

**Table 3.** Results from think-aloud testing.

Quote	Interpretation	Design implications [25,36]
Man 1: I was expecting [the virtual health coach in the app] to say something and she didn’t... then for her to say, “We’re excited about the progress that we’re making.” First... what progress?... we moved right away to come into the self care section and the scripting here seems to assume that someone is going to go through the topics in order... It seems out of context. Man 2: It should simply tell you. It should start at the beginning... She’s just making assumptions that we know... there needs to be a clear order of how to progress through the thing.	The participant expresses some confusion about the sequence of dialog as he goes through the educational segments. Later, other participants also describe a lack of clear progression from one subject to the next.	Older adults may have significant difficulty with mHealth^a^, especially when conversational agents do not communicate in natural conversation styles.
Man 2: Stress?... is that like environmental stress? Like outside? Working out? Traffic?	The virtual health coach describes the effect of stress on BP^b^, but the participant reports that the type of stress is not clear.	Stress mitigation is an important feature of smartphone apps to manage hypertension.
Woman 1: I think like the topics are helpful. They’re very related... And all of the stuff they talk about and each of the topics were also very relevant [to BP].	The participant appreciates that the included topics are useful and related to hypertension self-management.	—^c^
Woman 2: then this doctor was saying that like wait... is she a doctor? Man 3: And every time I hear that she learned something from somebody... I thought she was somebody that had credentials. I don’t know. Woman 2: I thought that she was a doctor. But then she’s not really dressed like a doctor.	Two participants discuss confusion about the virtual health coach’s credentials. They describe wanting to trust the coach but are unsure if she is trustworthy.	Transparency and trust are important, especially among racially minoritized or marginalized groups. Knowing the credentials of the person delivering information is key. Occupation-specific attire can improve credibility.
Man 3: I mean I guess you know this app is made for people to learn about the subject. So you know I feel like I could just like just get to the point. I could just Google the stuff too... you would have to be very motivated to stick with it.	The participant describes that if the goal is to gain knowledge, there are easier, less cumbersome ways to achieve that goal.	—
Man 2: She’s mentioning someone named Paul. We have no idea who Paul is so... Man 1: “Sometimes I am reminded that if Paul is not having symptoms, his blood pressure is still high.” And I should care because???	The participants describe confusion about parts of the virtual health coach’s dialogue. They express frustration that some parts of the conversation do not feel relevant.	Researchers and designers may have a perspective of what constitutes a valuable insight that differs from the perspective of the end user.
Man 3: I feel like the personal appearance of somebody to make them more relatable is pretty relative to the person using it though.	The participant describes the importance of cultural representation in the virtual health coach but recognizes that diverse end users would each require something different.	Physical attributes and conversation style could be optimized to reflect the end user. However, design experts suggest that trust and credibility are more likely gained from the system’s professionalism and knowledge.
Man 4: 138 over 88. I mean I know it’s high. It’s definitely elevated. But what does this mean and what does that mean? That’s what I always wanted to know... If like she says your blood pressure is elevated what should you do about it? Woman 2: Sometimes I wonder what the numbers mean. I think it’s cool to know, to have that information.	The participants react to their BP readings and express not understanding what the numbers mean.	The mHealth system should provide suitable education, personal feedback, and interaction reflective of disease severity. If critical findings occur, there should be a mechanism to communicate with relevant medical professionals.
Man 5: But it’s about access. It’s a 100 dollar thing [BP monitor]. Man 1: Fixed income. You gonna beat yourself and pay for pills or buy a new monitor?	The participants describe potential difficulty with high costs and a lack of access to mHealth tools in addition to the requirements such as medication.	Barriers to accessing technology can lead to a “digital divide” in which people from marginalized backgrounds are not able to benefit from mHealth. Technological advances should be designed for easy implementation into various communities.

a mHealth: mobile health.

b BP: blood pressure.

c Not applicable.

**Figure 4.**
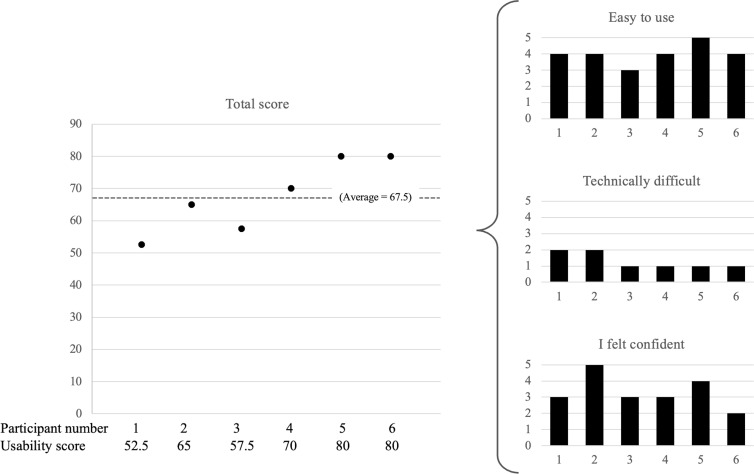
Summary of usability results. Participants found the tool to be about average in overall usability. Specifically, the tool was rated as easy to use, with low technical difficulty. Participants ranged in their confidence of using the intervention, with scores of 2 to 5, average 3.3. To calculate the total score for each respondent: for odd-numbered questions, subtract 1 from the response; for even-numbered questions, subtract the response from 5. Then, add the scores for all ten questions to calculate the total score out of 100 [38].

Furthermore, participants completed the “critiquing the system” activity. Figure 4 shows a summary of usability results from the 6 (75%) participants who completed the usability survey. Table 4 presents individual responses to the usability survey.

Overall, the group found the mHealth tool to be of average usability. Per the survey’s scoring system, total scores above 68 are considered “above average.” Our participants’ total scores ranged from 52.5 to 80, with a group average of 67.5 (SD 11.4). All participants rated the intervention as “easy to use.” Participants reported low technical difficulty and a moderate level of self-confidence in using the intervention.

**Table 4.** Individual participant results from system usability survey.

Question	Participant 1	Participant 2	Participant 3	Participant 4	Participant 5	Participant 6	Response^a^, mean (SD)
I think that I would like to use this system frequently.	2	4	3	3	4	4	3.3 (0.82)
I found the system unnecessarily complex.	3	2	3	1	2	1	2 (0.89)
I thought the system was easy to use.	4	4	3	4	5	4	4 (0.63)
I think that I would need the support of a technical person to be able to use this system.	2	2	1	1	1	1	1.3 (0.52)
I found the various functions in this system were well integrated.	3	3	4	3	4	4	3.5 (0.55)
I thought there was too much inconsistency in this system.	3	3	3	2	1	1	2.2 (0.98)
I would imagine that most people would learn to use this system very quickly.	2	4	3	3	4	4	3.3 (0.82)
I found the system very cumbersome to use.	3	4	3	3	4	1	3 (1.1)
I felt very confident using the system.	3	5	3	3	4	2	3.3 (1.03)
I needed to learn a lot of things before I could get going with the system.	2	3	3	1	1	2	2 (0.89)
Calculated total score	52.5	65	57.5	70	80	80	67.5 (11.4)

a To calculate the total score for each respondent: for odd-numbered questions, subtract 1 from the response; for even-numbered questions, subtract the response from 5. Then, add the scores for all 10 questions to calculate the total score out of 100 [38].

#### Understanding: Methods to Analyze Barriers to Ideal Intervention Use

**Figure 5.**
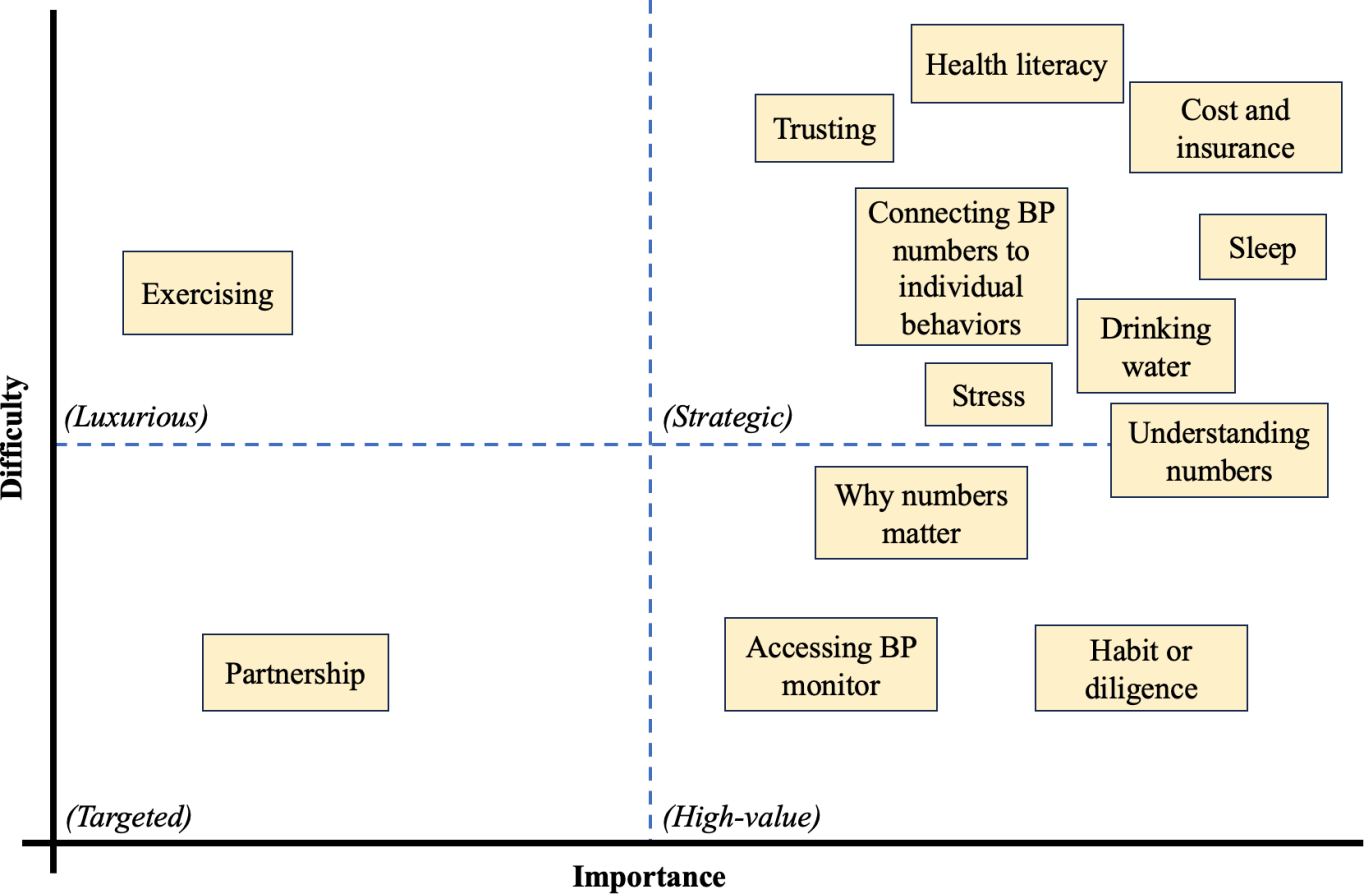
The importance and difficulty matrix*.* The axes represent the importance (high versus low) and difficulty (high versus low) of aspects of hypertension management. Four quadrants are labeled as follows: low importance, low difficulty quadrant is labeled as “targeted” because these are the simple tasks that are achieved with little thought or effort. The upper left quadrant contains “luxurious” aspects that are costly with little return. For example, the group felt that *exercising* belonged in this high difficulty, low importance quadrant. The upper right features “strategic” aspects that produce big results via large effort. Finally, the “high value” quadrant features relatively lower effort items with high impact.

The “importance and difficulty matrix” activity encouraged participants to thoughtfully describe perceived patterns and priorities about hypertension management. During this activity, the moderator and participants used a whiteboard to create a poster with a 2×2 table. Four quadrants were drawn with axes representing the importance (high vs low) of each aspect of hypertension management, as well as difficulty (high vs low). Figure 5 is a reproduction of the original table created from the whiteboard and handwritten sticky notes. Participants identified *partnership* as one aspect of hypertension management that is both low difficulty and low importance. Participants discussed that partnership between patient and physician should be easily accomplished but did not feel that it was as important as other concepts that were identified by the group. This low importance, low difficulty quadrant is labeled as “targeted” because these are the simple tasks that are achieved with little thought or effort. The upper left quadrant contains “luxurious” aspects that are costly with little return. The group felt that *exercising* belonged in this high difficulty, low importance quadrant. The upper right features “strategic” aspects that produce big results via large effort. Finally, the “high value” quadrant features relatively lower effort items with high impact. Some complementary concepts were felt by the group to be of high value, including *trusting* the health care system, *health literacy*, and *understanding* the BP numbers. *Cost* of and *access* to hypertension management tools were also classified as highly important.

**Figure 6.**
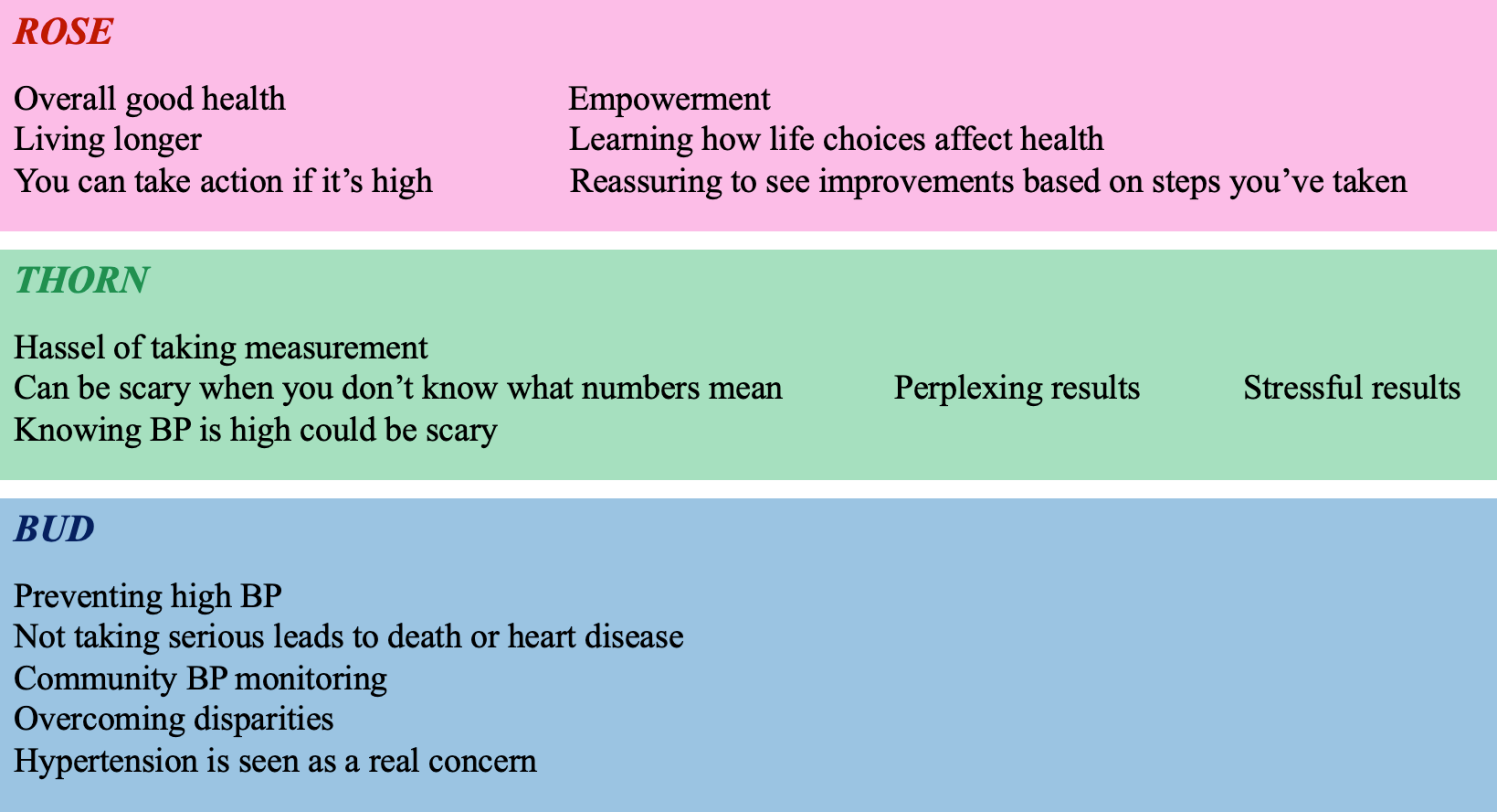
Rose, thorn, bud. The “rose, thorn, bud” activity encouraged participants to codify aspects of the hypertension intervention as being positive, negative, or as having promise (rose, thorn, or bud, respectively). BP: blood pressure.

The “rose, thorn, bud” activity further fostered prioritization of future design iterations. This activity encouraged participants to codify aspects of our hypertension intervention as being positive, negative, or as having promise, as shown in Figure 6. Participants felt that hypertension management would lead to positive outcomes such as better BP control, good health, and longevity. The group also identified that patients who use the intervention would be empowered to improve their health. Negative aspects of hypertension management related to the task of physically taking one’s BP and problems with not understanding the results. Important insights about the potential of our mHealth intervention were related to mitigating CVD risk and overcoming disparities at the community level.

#### Making: Methods to Design Future Iterations

As shown in rose, thorn, bud, participants agreed upon the benefit of the hypertension management intervention, and this final activity encouraged the group to further envision the possibilities of hypertension management with a future iteration of our mHealth intervention. The “cover story,” informed by the prior activities, resulted in the group envisioning mHealth as a conduit for marginalized communities to obtain a healthy lifestyle in the future. The activity asked participants to imagine a headline news story and create quotes, pictures, and text to convey a successful future of hypertension mHealth for Black patients in their community. Participants supported a shared vision in which Black residents of their city would be thriving and healthy. Their cover story was entitled, “Pittsburgh, Now a Livable City for Blacks.”

## Discussion

### Principal Findings

In this HCD study, community representatives found an existing mHealth hypertension app to be useful and revealed important elements to enhance the intervention’s future health equity outcomes. Facilitators to our intervention’s success included educational topics that were perceived as relevant to hypertension self-management. We also determined key findings for equitable mHealth design and implementation. Through HCD, we received feedback for future modification, specifically for stress management, community health, and improved design fidelity. This sequence of methods facilitated ideation, led to discussion of insightful feedback, refined design priorities, and helped with envisioning future solutions.

### Comparison With Prior Work

To date, educational features have been among the most studied mHealth hypertension self-management functionalities [42]. Our intervention included educational modules about understanding BP results, which participants noted to be useful and relevant. Future mHealth work should focus on educational opportunities to better facilitate an understanding of one’s BP results, as had repeatedly emerged in our study. However, as was also indicated in our study, educational features alone will not ensure user satisfaction. Experts recommend that technological interventions are not only educational but that they are also usable and accessible for the target population as has been shown for conditions such as hypertension, mental health, diabetes, and chronic pain [22,31]. According to our survey results, our current intervention was easy to use. Our participants reported low technical difficulty and a moderate level of self-confidence when using the intervention. A systematic review of mHealth hypertension management programs found that interventions can lead to BP control if they use multifaceted functions and messaging that are individually tailored to the needs of each patient [43]. Those authors recognized that further research about the cultural adaptation of interventions is needed [43]. Our findings help to fill the knowledge gap regarding patient-centered, community-engaged ways to test mHealth tools for usability and accessibility.

Although we did not measure device adherence in this study, others have shown that the value derived from using technology must be sufficiently high for patients to adhere to the intervention as part of their routine [44]. Defining patient-perceived value as it relates to hypertension deserves further study. As our results indicate, strategic next steps should prioritize features that are of high importance to end users. Design aspects that can build trust in the health care system, facilitate health literacy, and improve patients’ understanding of their BP will be important. Systematic reviews have shown that chronic disease management requires multimodal interventions that feature educational, tailored messages, interactive communication, and multifaceted functions [7,43]. The mHealth literature underscores the importance of digital health literacy, especially among vulnerable populations [45]. Data suggest that disproportionately affected and disadvantaged populations often lack the technological resources to keep pace with the rapid technological developments in health care [46]. Many mHealth programs require high literacy and are inaccessible for individuals with limited educational attainment or limited health and digital literacy [47]. Our participants felt that presenting information at an appropriate level for patients’ level of health literacy and providing education to improve hypertension knowledge were of central importance in an mHealth tool. Furthermore, our study revealed participant frustration over system redundancies and a perceived lack of flow between modules. A lack of app functionality integration could lead to intervention nonadherence, whereas platforms with seamless data integration may have high adherence [48]. Nevertheless, our group of users found the technology to be usable in its current state. Participants reported a moderate level of self-confidence in using our mHealth tool.

These findings suggest future technological tools may hold promise for marginalized patient populations. By involving participants from backgrounds that are often excluded from clinical trials, researchers can ensure that technological advances are applicable to broader audiences, thus potentially overcoming barriers to equitable hypertension management. mHealth self-management tools are best designed when their functionalities and modules are based on one or more theoretical models for behavior change [42]. Our theory-informed approach assisted our critique and understanding during the HCD session and bolstered our analyses with validated conceptual models of chronic disease management [49]. The Theory of Planned Behavior has been used to conceptualize hypertension management previously, especially among vulnerable patients such as older adults and women [40]. Other researchers have presented another dynamic behavior model that includes trust and lived experiences as determinants of BP management efforts [50].

Hypertension self-management interventions should provide clarity of what one’s BP results mean and how to respond to them in real time. Such features would likely lead to higher confidence in using mHealth, thus supporting patient autonomy as well as patient-clinician partnerships [51]. Key functionalities of hypertension self-management tools should incorporate communication with clinicians, possibly by exporting users’ data via a personal platform or patient portal that ensures privacy and security [42]. Others have reported the desire for patients to use mHealth to bridge the communication between patients and their clinicians in co-managing hypertension [24]. Interestingly, our group rated patient-clinician partnerships as a simple, basic component of hypertension management. While the literature supports mHealth as a tool for comanagement between clinicians and patients, all too often, minoritized, vulnerable groups do not achieve therapeutic partnerships with their clinicians to the same degree as White patients [52]. Standardized methods for handling accumulated BP readings and communicating the readings to clinicians are needed. Health care organizations and clinical institutions should invest in the development of mHealth platforms that can securely communicate with patients.

Our findings suggest that future hypertension mHealth tools should facilitate equitable BP management and hypertension care beyond the individual patient. Community-level interventions could include improved neighborhood access to digital, internet-based technology. Our group provided insights regarding future mHealth interventions to address hypertension disparities and mitigate heart disease risk at the community level. Community involvement with app design can positively impact the trust of minoritized participants and should be further explored to build meaningful mHealth interventions for hypertension and other conditions [25]. Furthermore, cultural knowledge and conversational idioms could enhance acceptance among marginalized groups [25]. Virtual agents, such as the one used in our study, use life-like features and attributes to enhance users’ perceptions of friendliness and empathy. Our findings suggest that details such as professional attire, title, and credentials had more influence on trust and credibility than did physical attributes. Future iterations could make the virtual health coach appear more racially inclusive and relatable, but professional characteristics should be emphasized as well. Design experts note that a clear designation of character roles is important. For example, physician-specific attire could increase participants’ perceptions of clinical credibility [25].

### Limitations

Limitations of this work include the small sample size. In this convenience sample, CAB member participation was limited by availability to attend the scheduled HCD session. However, HCD is primarily a qualitative, rather than quantitative, process. Thus, while a low number of participants limits statistical analysis, it was an ideal group size for HCD activities [37]. Another limitation is that not all participants had hypertension, and 3 of 8 had self-described prior health care knowledge, thus potentially limiting the generalizability of these findings to patients with hypertension. Experts have recommended that advisory boards reflect the community of interest, which can be demonstrated by shared identities, illnesses, experiences, histories, or cultures [53]. By these standards, our board was inclusive and representative. Finally, although our work was helpful in generating a discussion about digital equity, we were unable to fully assess implementation barriers to equitable digital technology use. For example, others have described the lack of affordable internet access and limited smartphone availability as barriers that disproportionately affect people from minority backgrounds [22]. Future work is required to better understand the cost and policy implications of equitable mHealth use for hypertension care.

### Conclusions

Our comprehensive, integrated approach to intervention development combined theory and evidence to ground our intervention in an in-depth understanding of the avenues for behavior change and facilitators specific to our participant demographics [12]. Future research should include pragmatic and implementation science methodologies to iteratively test new mHealth developments. The results showing “average” usability are important because they allow the development of future iterations that will later be compared to this baseline. Our findings emphasize that both high monetary cost and limited access to hypertension technologies create barriers to BP management. Therefore, policy implications include disseminating evidence-based mHealth that uses affordable technology and devices.

This study was a novel use case example of HCD as a patient-centered methodology to improve a hypertension management tool. Interest in HCD is growing in medical education and in global health innovation as a response to the needs of end users [54]. However, clinical researchers have not used HCD methods as readily. Our study is among a growing body of research to evaluate the impact of HCD for clinically relevant interventions. Despite our study’s limitations, we showed clear and actionable ways to improve future mHealth tools for hypertension. By regarding mHealth through an equity lens, clinicians, designers, and researchers can begin to move the field of chronic health management forward and implement successful strategies in the future.

## Abbreviations

BP: blood pressure
CAB: community advisory board
CVD: cardiovascular disease
FRAME: Framework for Reporting Adaptations and Modification to Evidence-Based Interventions
HCD: human-centered design
mHealth: mobile health
